# Electrospun Sandwich-like Structure of PVDF-HFP/Cellulose/PVDF-HFP Membrane for Lithium-Ion Batteries

**DOI:** 10.3390/molecules28134998

**Published:** 2023-06-26

**Authors:** Xingfu Zi, Hongming Wu, Jiling Song, Weidi He, Lu Xia, Jianbing Guo, Sihai Luo, Wei Yan

**Affiliations:** 1 Department of Polymer Materials and Engineering, College of Materials and Metallurgy, Guizhou University, Guiyang 550025, China; 13688582954@163.com; 2 National Engineering Research Center for Compounding and Modification of Polymer Materials, Guiyang 550014, China; whmand1988@sina.com (H.W.); songelin@126.com (J.S.); hwd3301932@163.com (W.H.); lrasyw@163.com (W.Y.); 3 Department of Chemistry, Norwegian University of Science and Technology (NTNU), 7491 Trondheim, Norway; lux@ntnu.no

**Keywords:** lithium-ion batteries, separator, cellulose, electrospinning, interfacial compatibility, cycle stability

## Abstract

Cellulose membranes have eco-friendly, renewable, and cost-effective features, but they lack satisfactory cycle stability as a sustainable separator for batteries. In this study, a two-step method was employed to prepare a sandwich-like composite membrane of poly(vinylidene fluoride-co-hexafluoropropylene) (PVDF-HFP)/cellulose/ PVDF-HFP (PCP). The method involved first dissolving and regenerating a cellulose membrane and then electrospinning PVDF-HFP on its surface. The resulting PCP composite membrane exhibits excellent properties such as high porosity (60.71%), good tensile strength (4.8 MPa), and thermal stability up to 160 °C. It also has exceptional electrolyte uptake properties (710.81 wt.%), low interfacial resistance (241.39 Ω), and high ionic conductivity (0.73 mS/cm) compared to commercial polypropylene (PP) separators (1121.4 Ω and 0.26 mS/cm). Additionally, the rate capability (163.2 mAh/g) and cycling performance (98.11% after 100 cycles at 0.5 C) of the PCP composite membrane are superior to those of PP separators. These results demonstrate that the PCP composite membrane has potential as a promising separator for high-powered, secure lithium-ion batteries.

## 1. Introduction

The resolution of resource crises and environmental pollution can be achieved through the development of green and renewable energy technologies [1,2,3]. To address this issue, many energy storage devices have been studied, such as ammonium-ion batteries [4] and metal-ion batteries [5,6,7]. One such technology is the lithium-ion battery, which is widely used due to its high capacity, lack of memory effect, low self-discharge, and stable output voltage [8,9,10]. However, lithium dendrites generated during charging and discharging cycles can easily pierce conventional polyolefin-based separators [11], making it crucial for separators to possess good electrolyte wettability, high mechanical strength, high thermal/dimensional/chemical stability, and superior electrochemical performance, especially for power batteries [12,13,14]. Due to their low porosity, intrinsic non-polar nature, and low melting points, conventional commercially available polyolefin-based separators are associated with unsatisfactory electrolyte wettability and thermal shrinkage at high temperatures, ultimately leading to battery safety accidents and impeding the electrochemical performance of lithium-ion batteries [15]. Furthermore, these separators are made from finite fossil fuels and are not renewable, biodegradable, or sustainable, thus contradicting green and energy-saving social objectives [16,17].

Cellulose, as one of the most abundant polymeric materials, has numerous advantages of biocompatibility, mechanical properties, renewability, and cost. For instance, its abundance of hydrogen bonds ensures excellent mechanical properties in cellulose membranes [18,19,20,21,22,23]. Xu et al. prepared a nanocellulose-carboxymethylcellulose gel electrolyte for aqueous zinc-ion batteries that exhibited excellent cycling stability and high ion conductivity [24]. Neeru et al. prepared a gel polymer electrolyte using nanocellulose and used it in a sodium-ion battery to achieve stable sodium deposition. This electrolyte had a high liquid-electrolyte absorption rate (up to 2985%) and high ion conductivity (2.32 mS cm^−1^) [25]. Du et al. [26] successfully developed a cellulose hydrogel with a tensile strength of 14.61 MPa by incorporating the crosslinking agent epichlorohydrin (ECH) into the cellulose solution. Nanoporous cellulose aerogels have also been prepared to enhance the interface compatibility between the membrane and the electrolyte [27,28]. Wan et al. [29] generated a cellulose aerogel using a dissolving-regenerating-supercritical drying method, creating a membrane with a nano-multi-hollow network structure and high porosity that significantly improves electrolyte absorption and ionic conductivity. While the hydroxyl groups in cellulose aid in lithium salt dissociation and promote ionic conductivity, the membrane’s high surface energy from these same groups can also impede lithium-ion migration, resulting in high interface impedance and poor cycle stability in lithium-ion batteries [30,31,32]. Therefore, developing cellulose membranes with improved interface stability is crucial for their practical application in lithium-ion batteries.

Electrospinning is a commonly employed method for producing separators with high porosity and large specific surface area for upgraded electrolyte uptake and ionic conductivity, which can enhance the electrochemical performance of lithium-ion batteries [33,34]. Various polymers have been used for preparing separators by electrospinning, including polyvinylidene fluoride (PVDF) [35], polyethylene oxide (PEO) [36], polyacrylonitrile (PAN) [37], and poly(vinylidene fluoride-co-hexafluoropropylene) (PVDF-HFP) [38]. PVDF-HFP membrane is preferred due to its high dielectric constant, low surface energy, and superior resistance to chemical degradation owing to its C-F chemical bond [39]. However, electrospun PVDF-HFP fibrous separators suffer from several drawbacks such as low mechanical strength, poor dimensional stability, and high thermal shrinkage, resulting in poor electrolyte retention and low ionic conductivity [40,41].

In this study, a sandwich-like composite membrane separator consisting of PCP was fabricated using a two-step method, which involved first dissolving and regenerating a cellulose membrane and then electrospinning PVDF-HFP on its surface. The resulting PCP composite membrane exhibits outstanding properties such as high porosity (60.71%), good tensile strength (4.8 MPa), and thermal stability up to 160 °C. Furthermore, it shows excellent electrolyte uptake properties of 710.81 wt.%, low interfacial resistance (241.39 Ω), and high ionic conductivity (0.73 mS/cm) compared to commercial polypropylene (PP) separators, which have interfacial resistance of 1121.4 Ω and ionic conductivity of 0.26 mS/cm. The rate capability (163.2 mAh/g) and cycling performance (98.11% after 100 cycles at 0.5 C) of the PCP composite membrane are also superior to those of PP separators. The electrospun PVDF-HFP layers prevent the hydroxyl groups on the cellulose membrane from contacting the metal lithium, which reduces the interfacial impedance between the membrane and electrode. The cellulose film functions as an intermediate layer to support the PVDF-HFP film, thereby enhancing the mechanical properties of the composite film and improving the poor dimensional stability and high thermal shrinkage of the PVDF-HFP film.

## 2. Results and Discussion

### 2.1. Surface Morphology of Membranes

**Figure 1:**
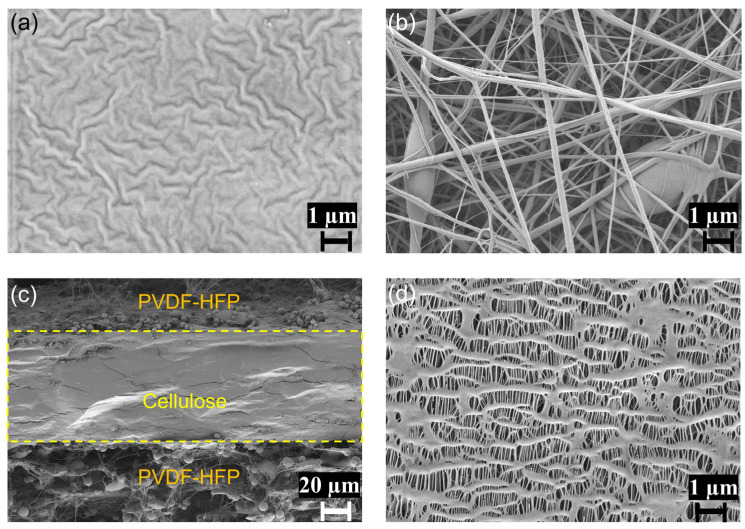
SEM images of cellulose (**a**); PCP composite membrane surface morphology (**b**); cross-section of PCP membrane (**c**) showing a sandwich structure; commercial PP membrane surface morphology (**d**).

Figure 1a–c shows the SEM images of the cellulose membrane (CM) and PCP membrane. The CM has dense surface morphology (shallow wrinkles), as shown in Figure 1a. No apparent holes or pores were observed on the CM, which can be attributed to the strong hydrogen-bond interaction of the cellulose molecules. In contrast, the PCP composite membrane is composed of individual nanofibers, as displayed in Figure 1b. The fiber interlocking on the PCP surface provides higher porosity, which is a crucial factor in the electrolyte uptake rate, while the dense surface of the CM leads to less desirable porosity. The cross-sectional SEM clearly reveals the sandwich structure of the PCP (see Figure 1c). The tiny spherical particles in the SEM image resulted from the quenching of the liquid nitrogen and some of the polymer from the spun liquid. As a comparison, Figure 1d shows a commercial PP membrane separator, also showing the presence of nanopores on the surface of the membrane.

### 2.2. Electrolyte Uptake Performance

**Figure 2:**
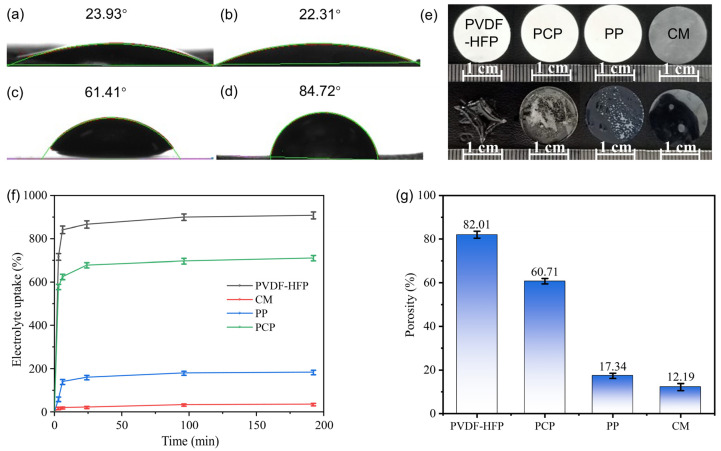
Contact angles of different membranes measured using liquid electrolyte: (**a**) PVDF-HFP; (**b**) PCP; (**c**) PP; (**d**) CM; (**e**) photographs of different membranes before and after uptake of electrolyte; electrolyte uptake behaviors (**f**) and porosities (**g**) of different membrane samples.

The wettability of a separator plays a critical role in determining its ionic conductivity and electrochemical performance, which in turn affects the overall battery performance. Figure 2a–d presents the contact angles of all the different samples, with the PCP and PVDF-HFP membranes having almost identical contact angles of approximately 23°. This could be due to the high specific surface area of the PCP surface, resulting from its interwoven fiber structure, which promotes electrolyte affinity [42]. In contrast, the CM has the highest contact angle of 84.72°, which may be attributed to its dense surface. It is worth noting that the pure PVDF-HFP membrane experienced severe curling and shrinkage after the electrolyte was removed (see Figure 2e), indicating the instability of the membrane in the presence of electrolytes.

Furthermore, Figure 2f,g show the electrolyte uptake rate and porosity of the samples. All samples exhibited a similar electrolyte uptake behavior, with an initial rapid increase followed by a plateau. The final electrolyte uptake rate was the highest for the PVDF-HFP membrane (908.33 wt.%), followed by the PCP membrane (710.81 wt.%), the PP membrane (184 wt.%), and the CM (36.36 wt.%). The higher electrolyte uptake rate of the PCP membrane can help to reduce internal resistance and increase the ionic conductivity of the lithium battery [43]. The PCP membrane had the highest porosity due to its staggered cross-structure, but it is lower than the pure PVDF-HFP membrane because of the lower porosity of the CM interlayer. These results suggest that the microstructure and porosity of the membrane have a significant impact on its wettability.

### 2.3. Surface Analysis

The surface properties of a separator have a significant impact on the internal resistance of a battery. The FTIR spectra of CM and PCP are presented in Figure S1a Supplementary Materials, with the CM sample showing peaks corresponding to the vibration of O-H bonds in hydroxyl groups at around 3342.03 cm^−1^ [31]. Previous studies have demonstrated that the hydroxyl groups on the surface of a cellulose separator can react with the electrode and increase the internal resistance of a lithium battery [32]. In contrast, the O-H vibrational peak in the hydroxyl group was not observed in the PCP and PVDF-HFP samples. This is attributed to the presence of PVDF-HFP nanofibers on the PCP surface, which hinder the hydroxyl groups. Energy-dispersive spectroscopy (EDS) mapping measurements depicted in Figure S1b,c revealed that the element O content on the PCP surface is significantly lower than that on the CM surface, confirming the absence of hydroxyl groups on the PCP surface.

Table 1 and Table 2 present the element contents on the surfaces of different samples, indicating that the CM and PCP samples have different element compositions. Specifically, the element O content of the PCP sample is only 1.76%, whereas that of the CM sample is 44.05%. The main surface elements of the PCP sample are C and F, which is consistent with the major elements contained in PVDF-HFP. The element O on the PCP surface is derived from the interlayer cellulose film. Meanwhile, Na and Cl elements may be residues of solvents in the cellulose membrane. The absence of hydroxyl groups on the PCP surface is further supported by the element content table, suggesting that the PCP has a more stable chemical structure.

### 2.4. Mechanical Properties of Membranes

**Figure 3:**
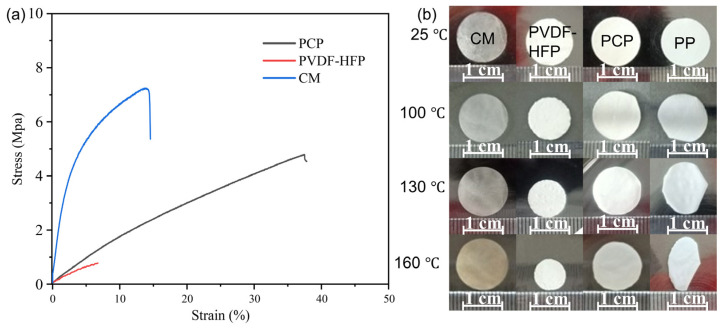
Stress-strain curves of different membranes (**a**); photographs of different membranes subjected to thermal shrinkage at different temperatures (**b**).

The separator serves not only as a physical barrier to prevent internal short circuits but also as a conduit for lithium-ion transport in the battery, making the mechanical properties of the separator essential to the battery’s performance [44]. Figure 3a shows the stress-strain curves for the PCP, PVDF-HFP, and CM membranes. The CM exhibits the highest tensile strength (7.2 MPa), which is significantly greater than that of the PCP (4.8 MPa) and PVDF-HFP (0.77 MPa) samples. The cellulose layer enhances the tensile strength of the sandwich-like structure of the PCP membrane. The thermal shrinkage of all membrane samples at different temperatures is shown in Figure 3b. The dimensions of the CM and PCP membranes remain constant even at a temperature of 160 °C, which is consistent with thermogravimetric (TG) curves (see Figure S1d). However, the PVDF-HFP sample and the commercial PP separator exhibit significant thermal shrinkage. Notably, the PCP membrane maintains dimensional stability at high temperatures due to the excellent properties of the cellulose layer in the composite membrane.

### 2.5. Electrochemistry Properties of Membranes

Ionic conductivity is a crucial parameter for electrolytes, as it describes the movement of ions in electrochemical reactions. Higher ionic conductivity leads to improved electrochemical reactions and reduced internal resistance. Typically, a coin battery (SS/SE/SS) with a separator (SE) is sealed using two identical stainless steel (SS) sheets. By subjecting the battery to electrochemical impedance spectroscopy, the ionic conductivity of the electrolyte can be estimated using Equation (3), *σ* = *L*/(*R_b_* ∗ *S*), where *R_b_* represents the bulk resistance and is defined by the intercept of the line in the electrochemical impedance spectroscopy (EIS) with the *x*-axis. Figure S2 shows the EIS of a coin battery (SS/SE/SS) with various separators, suggesting that the contact resistance of the CM is the highest. The PCP membrane has only half the resistance of the CM, despite being thicker. Table 3 shows the ionic conductivity of different samples calculated using Equation (3). The PCP membrane exhibits the highest ionic conductivity at room temperature, reaching 0.73 mS/cm, while the CM and PP membranes have values of 0.21 mS/cm and 0.26 mS/cm, respectively (the thickness of each layer of the membrane is 265 μm, 100 μm, and 265 μm, respectively). This increase in ionic conductivity may be due to the superior electrolyte retention ratio of the PCP composite, which enhances the transference of lithium ions and impedes hydroxy interaction.

**Figure 4:**
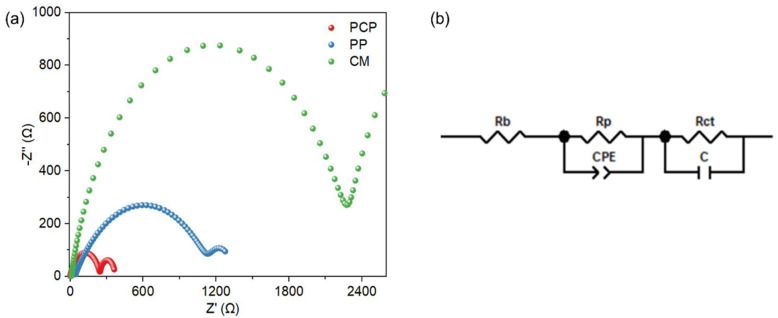
Nyquist plots of Li/SE/Li batteries using different separators (**a**); equivalent circuit diagram (**b**).

The Nyquist plots of Li/SE/Li coin batteries s with various separators are depicted in Figure 4, along with the corresponding circuit diagram. It is evident that the PCP sample shows the lowest bulk resistance. The CM has the highest interface resistance, which is significantly greater than that of the commercial PP and PCP membranes. This substantial resistance of the CM may be attributed to the hydrogen activity on the cellulose surface, which hinders the transport of lithium ions. In contrast, the PCP possesses the lowest interface resistance among all membranes, which may be attributed to two main causes: (1) the polar cellulose molecules enhance the compatibility of the electrolyte, resulting in increased electrolyte retention and wettability; and (2) the electrospun PVDF-HFP layer suppresses the activity of surface hydrogen, reducing the interface resistance of the PCP composite membrane.

**Figure 5:**
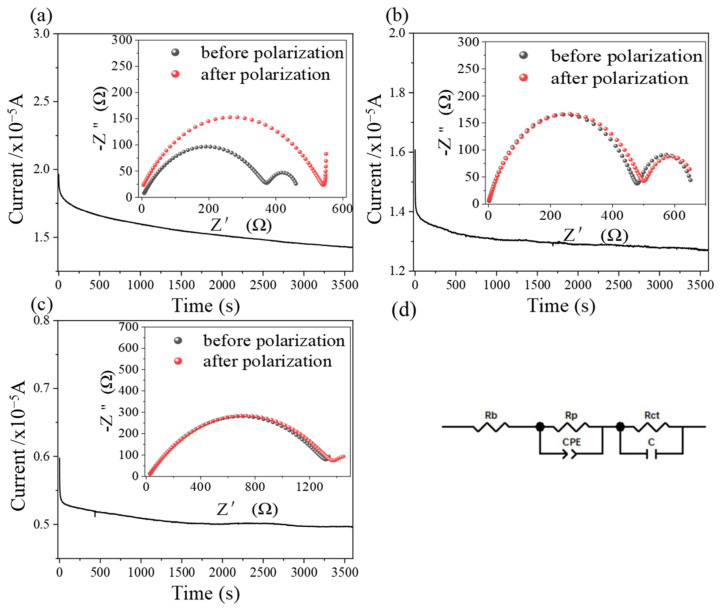
Current and time curves of DC polarization and Nyquist-plots before and after polarization using different membranes as separators: (**a**) PCP; (**b**) PP; (**c**) CM; (**d**) equivalent circuit diagram.

The lithium-ion transference number of the electrolyte is a critical performance parameter that affects battery properties. Figure 5 depicts the current-time curves of DC polarization and Nyquist plots before and after polarization for various separators. The resistance of the separators before and after polarization is used to calculate the lithium-ion transference number of the membranes using the equation $t_{{Li}^{+}}=\frac{I_{S}(\Delta V-I_{0}R_{0})}{I_{0}(\Delta V-I_{S}R_{S})}$, where $t_{{Li}^{+}}$ represents the Li ion transference number, *I_S_*, *I*_0_, *R_S_*, and *R*_0_ are the current and interfacial resistance after and before polarization, respectively, and Δ*V* is the polarization voltage set to 10 mV. The Nyquist plots of the membranes indicate that all batteries with different separators exhibit similar internal bulk resistance. All samples have an equivalent circuit, suggesting that lithium transfer in those samples occurs according to similar principles. The CM has the highest resistance, while the PCP exhibits the lowest resistance among all samples.

The lithium-ion transference number of the electrolyte is a crucial electrochemical performance value for the membrane, as it indicates the number of transferable Li+ ions at a given lithium-ion density. A higher lithium-ion transference number improves the charge transfer efficiency within the lithium-ion battery. Table 4 displays the calculated lithium-ion transference numbers of the membranes at room temperature. It can be observed that the PCP sample exhibits the highest *t*_Li+_ at 0.91, while the commercial PP separator and the CM only have values of 0.5 and 0.55, respectively. The excellent transference number can be attributed to the electrospun PVDF-HFP layer on the surface of the cellulose, which hinders the hydrogen activity of the cellulose, allowing for easy transport of abundant lithium ions in the PCP membrane.

The electrochemical stability of different membranes as separators was characterized by linear sweep voltammetry (see Figure S3). The CM and PP separators have electrochemical windows of 4.27 V and 4.45 V, respectively. However, the PCP separator exhibits the highest electrochemical stability among all the samples, with an electrochemical window of 4.61 V. This superior performance can be attributed to the unique sandwich-like structure of the PCP membrane, which effectively suppresses the activity of residual hydroxyl groups on the cellulose membrane.

### 2.6. Battery Performance

**Figure 6:**
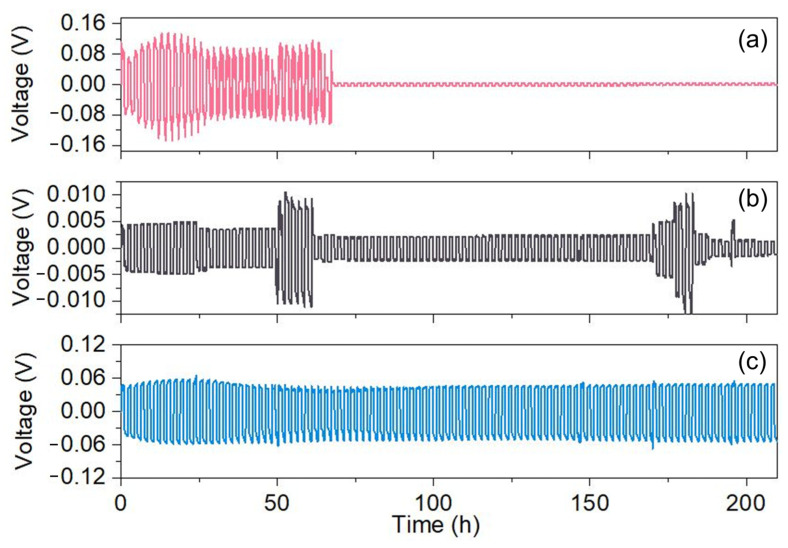
Cycle performance of the Li/SE/Li batteries at constant current density using different membranes as separators: (**a**) PP; (**b**) CM; (**c**) PCP.

The cycle stability of separators was evaluated by conducting cycle performance testing of the Li/SE/Li batteries using different separators at a constant current density. The batteries were charged at 0.05 mA (0.044 mA/cm^2^) for 1 h, followed by discharge at the same current density for 1 h after a 2-min pause. As shown in Figure 6, the PCP-based battery remained stable for 200 h, while the CM and PP batteries showed a decline after 60 h. These results indicate that the PCP sample has excellent cycle stability.

**Figure 7:**
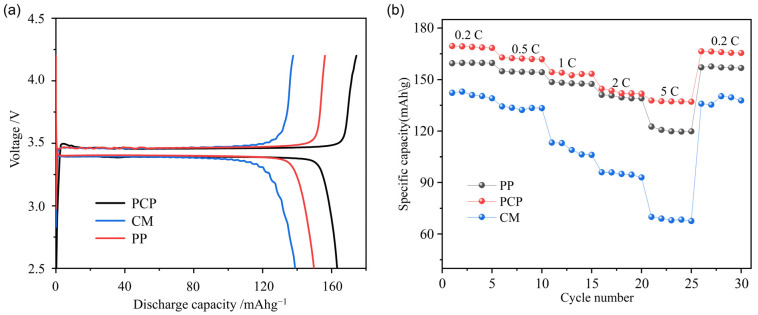
Charge and discharge curves (**a**) and rate performance (**b**) of Li/SE/LiFePO_4_ half-batteries using different membranes.

Figure 7a shows the charge-discharge curves and rate behavior of batteries using different membranes as separators. It can be observed that the battery using the PCP as a separator exhibits the highest discharge capacity among all the batteries. The capacity of the PCP battery is 163.2 mAh/g, while the PP battery and CM battery only have capacities of 149.7 mAh/g and 138.8 mAh/g, respectively. The capacity of the PCP battery is 17.6% higher than that of the CM. The improved charge-discharge performance of the PCP-based battery is due to the enhanced electrolyte uptake ratio and reduced interface resistance. Figure 7b shows the rate performance of the batteries using different membranes as separators. The PCP-based battery exhibits the best rate performance among all the batteries, which can be attributed to the high ionic conductivity and lithium-ion transference number of the PCP-based battery, thus reducing the internal bulk resistance of the battery.

**Figure 8:**
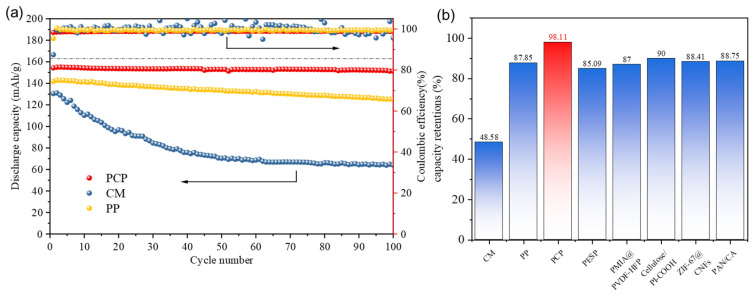
Cycle stability of different membranes as separators (**a**); comparison of capacity retention of PCP with other studies (**b**).

The cycle properties of batteries with different membranes as separators are presented in Figure 8. The PCP sample demonstrates the highest capacity retention (ratio of discharge capacity after the 100th cycle to the initial discharge capacity of the first cycle), at 98.11%, which is greater than that of the CM and PP samples, at 48.85% and 87.6%, respectively. Additionally, the PCP sample exhibits the best cycle stability compared to other membranes reported in the literature [45,46,47,48,49], as shown in Figure 8b. The excellent cycle stability can be attributed to two reasons: firstly, the large electrolyte retention of the membrane increases the transfer lithium ions number, and secondly, the PVDF electrospun layer on the surface of the cellulose hinders hydrogen activity, improving the interface compatibility with the electrode and leading to good cycle stability.

## 3. Materials and Methods

### 3.1. Materials

Cellulose powders (particle size 50 μm~100 μm, *M*_w_: 162.06), urea (AR, *M*_w_: 60.06), sodium hydroxide (NaOH, AR, *M*_w_: 40), epichlorohydrin (ECH, AR, *M*_w_: 92.52), and *N*-*N*-dimethlacetamide (DMAc, AR 99%, *M*_w_: 87.12) were purchased from Aladdin (Shanghai, China) without purification. The liquid electrolyte (1 M LiTFSI in DME: DOL = 1:1 vol% solution) was purchased from DoDoChem (Suzhou, China). Poly(vinylidene fluoride-co-hexafluoropropylene) (PVDF-HFP, average *M*_w_ ~400,000) was purchased from Macklin (Shanghai, China).

### 3.2. Methods

#### 3.2.1. Preparation of Cellulose Membrane

To prepare a 4.5% (by weight) cellulose solution, 2.25 g cellulose powder was added to a 50 mL solution of NaOH/urea/H_2_O (7%/12%/81%, weight/weight/weight) and the mixture was then stirred until the cellulose powder was fully dissolved. Next, 5 wt.% ECH was added into the cellulose solution and uniformly mixed by stirring for 20 min to acquire solution A. Solution A was then poured into a PTFE mold and dried at 60 °C for two hours to obtain a cellulose film.

#### 3.2.2. Preparation of Sandwich-Like Structure of PCP Composite Membrane

**Figure 9:**
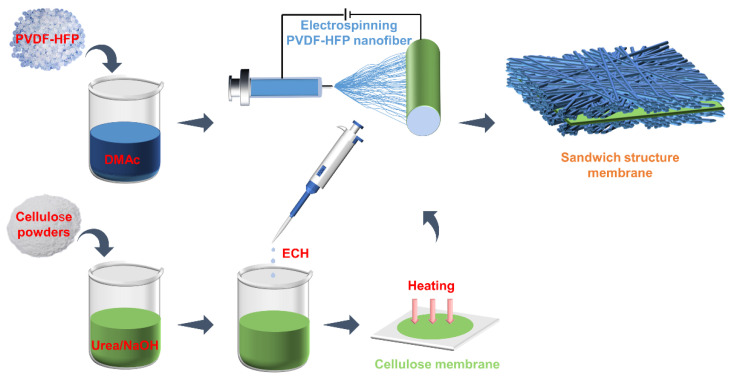
Schematic illustration of the preparation of PVDF-HFP/Cellulose/PVDF-HFP (PCP) composite membrane as a separator.

Firstly, 4.75 g PVDF-HFP was added to the DMAc to obtain 20 mL of PVDF-HFP solution (20 wt.%). The solution was then electrospun onto both sides of the cellulose by electrospinning (JDF05 Nano apparatus, Changsha, China) to produce a PCP composite membrane (see Figure 9). Electrospinning parameters were as follows: spinning voltage 15 kV, flow rate 1 mL/h, and spinning distance 15 cm. A PDVF-HFP membrane was also prepared under the identical conditions as a reference sample.

### 3.3. Characterization

#### 3.3.1. Microstructure and Morphology of Membranes

Firstly, the samples were spray-coated with a thin gold layer to facilitate imaging the microstructure of the samples. A cross-section of the PCP was obtained by immersing it in liquid nitrogen for several minutes and then breaking it off. The surface morphology of the membranes was confirmed using a scanning electron microscope (SEM, Quanta FEG250, FEI) with an acceleration voltage of 10 kV.

#### 3.3.2. Fourier Transform Infrared (FTIR) Spectroscopy Analysis

FT-IR spectra in the absorbance mode, over wavenumbers ranging from 400 to 4000 cm^−1^, of membranes with the same thickness were obtained using a Bruker (Karlsruhe, Germany) FT-IR spectrometer.

#### 3.3.3. Thermal Property of Membranes

The thermal properties of the membranes were evaluated by thermogravimetric analysis (TGA). Samples weighing 20 mg were weighed and heated from 40 °C to 450 °C at a rate of 10 °C per minute in a nitrogen atmosphere.

#### 3.3.4. Electrolyte Uptake of Membrane

The membranes were placed into an argon-filled glovebox and immersed in a liquid electrolyte. The amount of electrolyte uptake was determined by measuring the change in weight as the membranes swelled over a 48 h period, and was calculated using Equation (1):

(1) $$\eta =(w_{1}-w_{0})/w_{0}$$

where η is the swelling ratio and *w*_0_ and *w*_1_ represent the weight of the membrane sample before and after swelling in liquid electrolyte, respectively.

#### 3.3.5. Contact Angle Measurement

Contact angle measurements were carried out using a Kruss DSA25S (Kruss GmbH, Hamburg, Germany) contact angle goniometer at room temperature.

#### 3.3.6. Porosity

The masses of the membranes were measured before and after immersing in anhydrous ethanol for 3 h. The porosity (*p*) was then calculated using Equation (2), where *W_d_* and *W_w_* denote the mass of the membrane before and after immersion, *ρ_b_* is the density of anhydrous ethanol, and *ρ_p_* is the density of the membrane.

(2) $$p=\frac{{(w}_{w}-w_{d})/\rho_{b}}{{(w}_{w}-w_{d})/\rho_{b}+w_{d}/\rho_{p}}$$

### 3.4. Electrochemical Characterization

#### 3.4.1. Ionic Conductivity

**Scheme 1:**
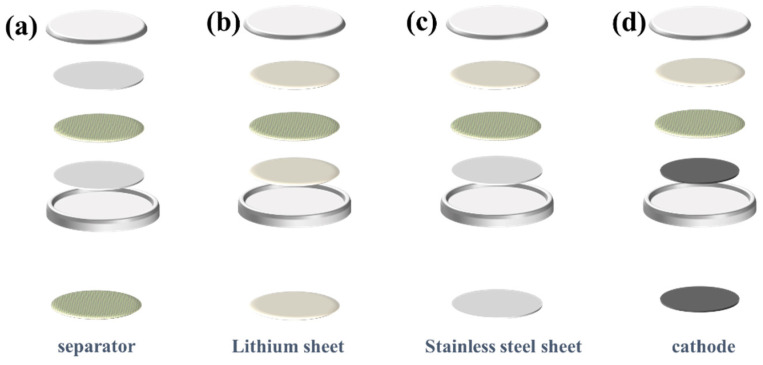
Coin battery structures: (**a**) stainless steel/separator/stainless steel; (**b**) lithium/separator/lithium; (**c**) lithium/separator/stainless steel; (**d**) lithium/separator/cathode.

Before assessing the ionic conductivity, the gel electrolyte samples were assembled into coin batteries with two stainless steel sheets as positive and negative electrodes (Scheme 1a). The ionic conductivity was measured by electrochemical impedance spectroscopy (EIS) between 0.01 and 10^6^ Hz with an amplitude of 10 mv. The final value was calculated using following Equation (3):

(3) $$\sigma =L/(R_{b}*S)$$

where *σ* is the ionic conductivity, *L* (cm) represents the thickness of the electrolyte, *S* (cm^2^) denotes the effective overlap area of the electrode and the electrolyte, and *R_b_* is the electrolyte bulk resistance.

#### 3.4.2. Lithium-Ion Transference Number

Before evaluating the lithium-ion transference number, the samples were assembled into coin batteries with a pair of lithium electrodes as the positive and negative electrodes (Scheme 1b). The lithium-ion transference numbers were obtained through EIS and chronoamperometry, and then calculated using Equation (4):

(4) $$t_{{Li}^{+}}=\frac{I_{S}(\Delta V-I_{0}R_{0})}{I_{0}(\Delta V-I_{S}R_{S})}$$

where $t_{{Li}^{+}}$ is the Li ion transfer number, *I_S_*, *I*_0_, *R_S_* and *R*_0_ are the current and interfacial resistance after polarization and before polarization, respectively, and ΔV is the polarization voltage set to 10 mV.

#### 3.4.3. Interfacial Compatibility

The interfacial compatibility was obtained by assessing a coin battery consisting of a pair of lithium electrodes and a gel electrolyte (Scheme 1b). EIS testing was conducted on the 7th day.

#### 3.4.4. Electrochemical Stability

The electrochemical stability was evaluated by assembling a coin battery consisting of lithium as the negative electrode and stainless steel as the positive electrode (Scheme 1c). Voltage was determined by linear sweep voltammetry (LSV), with voltages ranging from −0.5 V to 5 V at a scan rate of 1 mV/s.

### 3.5. Battery Performance

The coin battery (LiFePO_4_/GE/Li) with a metal lithium sheet as the negative electrode and LiFePO_4_ as the positive electrode was assembled (Scheme 1d). The battery performance was evaluated using a charge/discharge apparatus (BTS-5V10mA, NEWARE, Shenzhen, China), and the cut-off voltage was set at to 2.5–4.2 V.

## 4. Conclusions

In this study, a sandwich-like structure of a PVDF-HFP/cellulose/PVDF-HFP (PCP) composite membrane as a separator was prepared. First, the cellulose membrane was obtained through a dissolution-regeneration process. Next, the PVDF-HFP was electrospun onto the cellulose surface to produce the PCP composite membrane with excellent properties, such as high porosity (60.71%), good tensile strength (4.8 MPa), and thermal stability up to 160 °C. It also has exceptional electrolyte uptake properties (710.81 wt.%). The excellent electrochemical properties of the PCP membrane as a separator enabled the Li/PCP/LiFePO_4_ battery to achieve exceptional performance, with a discharge capacity of 163.2 mAh/g at 0.5 C, which was higher than that of the battery using PP as a separator at 149.7 mAh/g. Additionally, the composite membrane battery showed excellent cycle stability, with a capacity retention of 98.11% after 100 cycles. The exceptional discharge capacity, rate performance, and cycle stability were attributed to the excellent electrolyte retention capacity and sandwich-like structure of the PCP membrane. Given its remarkable performance, the PCP composite membrane prepared in this study has great potential for use as a separator in lithium-ion batteries, as it can significantly improve their cycle stability and safety features.

## Figures and Tables

**Table 1:** Elements composition on CM surface.

Element	wt.%	wt.% Sigma	At%
C	23.23	0.19	31.15
N	4.22	0.19	4.86
O	44.05	0.16	44.34
Na	27.24	0.11	19.08
Cl	1.25	0.02	0.57

**Table 2:** Element composition on PCP surface.

Element	wt.%	wt.% Sigma	At%
C	45.84	0.19	57.09
O	1.76	0.09	1.64
F	52.4	0.18	41.26

**Table 3:** Ionic conductivities of different membranes.

Membrane	Thickness (μm)	*σ* at 25 °C (mS/cm)
PCP	630	0.73
CM	100	0.21
PP	30	0.26

**Table 4:** Lithium transference number of membranes at room temperature.

Membranes	*R*_0_	*R_S_*	*I*_0_ (10^−5^ A)	*I_S_* (10^−5^ A)	*t*_Li+_
PCP	363.36	540.97	1.97	1.43	0.91
PP	477.9	499.79	1.61	1.27	0.50
CM	1316	1369	0.60	0.50	0.55

## Data Availability

The data presented in this study are available from the corresponding author upon reasonable request.

## Supplementary Materials

The following supporting information can be downloaded at https://www.mdpi.com/article/10.3390/molecules28134998/s1: Figure S1 FT-IR spectra of CM, PVDF-HFP and PCP membranes (a) and associated EDS mapping of O element in CM (b) and PCP (c), respectively. TG curves of different membranes (d); Figure S2 Interface impedance plots of different membranes. Figure S3 Linear sweep voltammogram of different membranes as separators.

## Sample Availability

Not available.
