# Supplementary material for: Electrospun Sandwich-like Structure of PVDF-HFP/Cellulose/PVDF-HFP Membrane for Lithium-Ion Batteries
Source: Molecules. 2023 Jun 26;28(13):4998. doi: 10.3390/molecules28134998 (PMC10343732; doi:10.3390/molecules28134998)
Supplement: Supplementary file 1 [file molecules-28-04998-s001.zip › molecules-2397996-supplementary.pdf]

Supporting Information

Electrospun sandwich-like structure of PVDF-HFP/cellulose/PVDF-HFP membrane for lithium-ion batteries

Xingfu Zi [1], Hongming Wu [2], Jiling Song [2], Weidi He [2], Lu Xia [3], Jianbing Guo [1,2,*], Sihai Luo [3,*] and Wei Yan [2]

[1] Department of Polymer Materials and Engineering, College of Materials and Metallurgy, Guizhou University, Guiyang 550025, China; 13688582954@163.com
[2] National Engineering Research Center for Compounding and Modification of Polymer Materials, Guiyang 550014, China; whmand1988@sina.com (H.W.); songelin@126.com (J.S.); hwd3301932@163.com (W.H.); lrasyw@163.com (W.Y.)
[3] Department of Chemistry, Norwegian University of Science and Technology (NTNU), 7491 Trondheim, Norway; lux@ntnu.no
* Correspondence: guojianbing_1015@126.com (J.G.); sihai.luo@ntnu.no (S.L.)

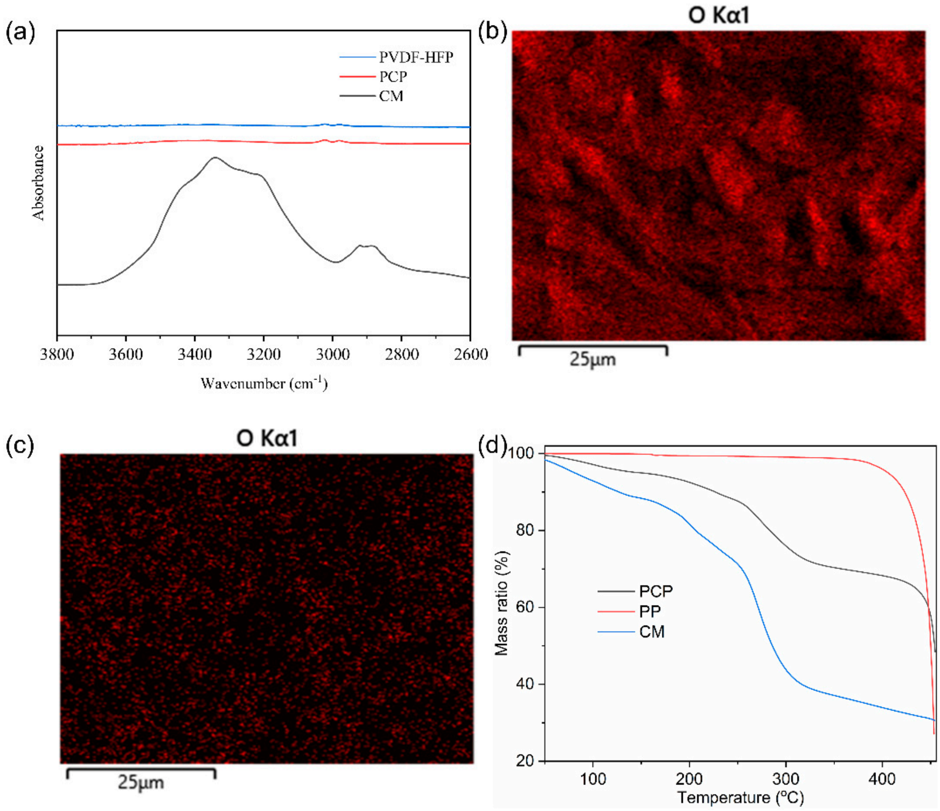

Figure S1 FT-IR spectra of CM, PVDF-HFP and PCP membranes (a) and associated EDS mapping of O element in CM (b) and PCP (c), respectively. TG curves of different membranes (d).

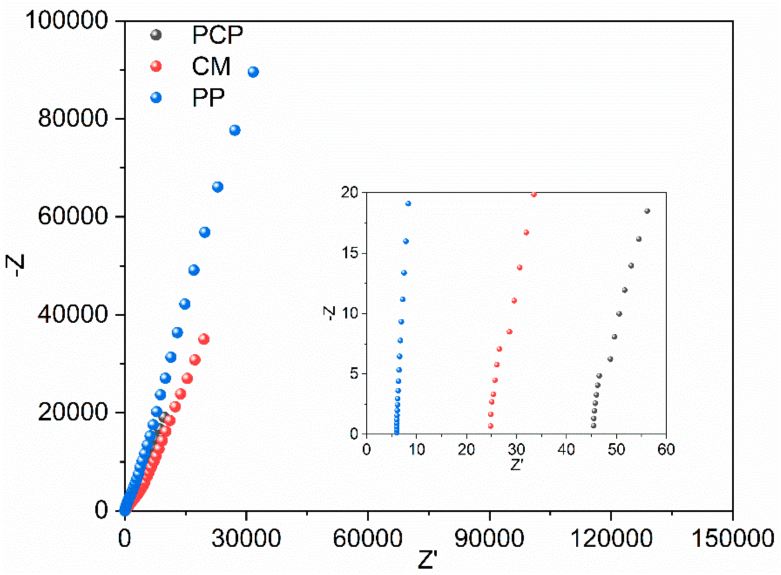

Figure S2 Interface impedance plots of different membranes.

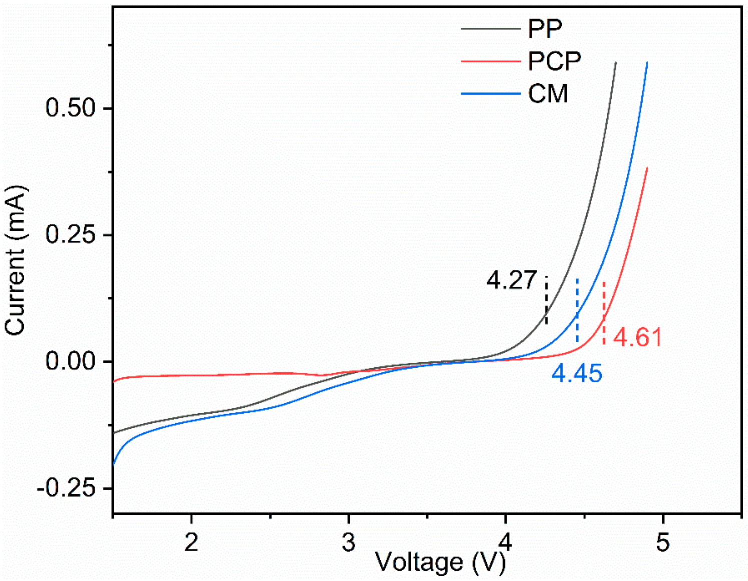

Figure S3 Linear sweep voltammogram of different membranes as separators.
